# Responsible antibiotic use labeling and consumers’ willingness to buy and pay for fluid milk

**DOI:** 10.3168/jds.2022-21791

**Published:** 2022-11-01

**Authors:** Robert Charles Schell, Ece Bulut, Hannah Padda, Amelia Greiner Safi, Paolo Moroni, Renata Ivanek

**Affiliations:** 1 Department of Population Medicine and Diagnostic Sciences, College of Veterinary Medicine, Cornell University, Ithaca, NY 14853; 2 Division of Health Policy and Management, School of Public Health, University of California–Berkeley 94720; 3 Department of Communication, College of Agriculture and Life Sciences, Cornell University, Ithaca, NY 14853; 4 Dipartimento di Medicina Veterinaria e Scienze Animali, Università degli Studi di Milano, Via dell’Università, 6, 26900 Lodi, LO, Italy

**Keywords:** antibiotics, willingness to pay, dairy milk, experimental auction, survey

## Abstract

Concerns about antibiotic resistant infections in the United States have called for reduction of antibiotic use in livestock, including dairy cattle. Although effective in curbing antibiotic use, universal organic dairy farming would be impractical and unattainable due to its high land and premium demands. The US Department of Agriculture’s organic certification, which completely eliminates antibiotic use in milk production, also raises animal welfare concerns, as it could discourage the use of antibiotics even to treat indicated diseases. Therefore, a proposed alternative for US consumers is a label indicating the responsible antibiotic use (RAU) – not complete elimination – that would minimize antibiotics more than conventional (unlabeled) milk and maximize animal welfare more than organic milk. Our goal was to determine consumers’ (1) self-reported preference and (2) willingness to pay for this hypothetical RAU label of milk relative to existing substitutes in organic and unlabeled fluid milk. We conducted (1) a nationally representative survey of US adults and (2) a randomized non-hypothetical experimental Becker-Degroot-Marschak auction with real money and real milk. Although almost half of the survey participants (48.5%) responded that they would buy a RAU-labeled milk, consumers in the experimental auction refused to pay a significant premium for the milk compared with unlabeled milk (mean willingness to pay (95% confidence interval) per half-gallon: $1.92 ($1.65–$2.19) for RAU-labeled milk versus $1.86 ($1.58–$2.13) for unlabeled milk). These results suggest that consumers’ survey-identified preferences for RAU-labeled milk could reflect either social desirability bias or a genuine preference for which, however, consumers simply will not pay a significant premium. The study provides preliminary data for future exploration of marketability of the proposed RAU label in the United States and demonstrates the benefits of using complementary survey and experimental auction approaches to understand the potential market for a new dairy product.

## INTRODUCTION

The emergence of antibiotic resistant infections due to antibiotic overuse, including in animal agriculture, poses a looming threat to human, animal, and environmental health. Consequently, antibiotic use in all areas of animal agriculture, dairy farming included, has come under scrutiny. One proposed solution to antibiotic overuse is their complete elimination, as exemplified by the USDA Organic Certification label (USDA AMS, 2015). Although in the United States an organic dairy farmer may treat their cattle with antibiotics if needed, they cannot market products as organic from dairy cattle that have ever been given antibiotics (US Government Information, 2018). Farmers must remove such cows entirely from the organic operation by selling them either to a conventional dairy farm or for processing as non-organic meat, which has important economic ramifications for the farmer (Brock et al., 2021).

Some evidence exists that the stringency of organic farms’ antibiotics-free requirements in the United States unintentionally incentivizes reluctance among organic farmers to treat diseases that could easily be cured by antibiotics, which leads to animal welfare concerns (Harðarson, 2002; Lund and Algers, 2003; Habing et al., 2016; Karavolias et al., 2018). Additionally, organic farms have strict rearing requirements, which include feeding animals with 100% organic feed, raising animals without the use of hormones, as well as pasture access and minimum land requirements (USDA ERS, 2016a); thus, a complete nationwide shift to organic practices could prove costly due to conversion cost (USDA ERS, 2016b) or environmentally devastating due to land use intensity (Borlaug, 2004).

Two groups of farmers exist in the United States, a small number of organic producers who raise animals without the use of antibiotics and a large number of conventional producers. Among conventional producers, some continue to overuse antibiotics with tactics such as blanket application of dry cow therapy, where even healthy cows receive treatment to prevent the new IMI (Ekakoro et al., 2018). Conventional farmers are generally skeptical of and show antipathy toward the organic movement because labeling and marketing of organic products reinforces consumers’ belief that organic farming is somehow “better” (Wemette et al., 2021). These views make widescale organic farming converts unlikely, but conventional farmers can still enact changes to lower their antibiotic footprint. For example, blanket antibiotic treatments of clinical mastitis have been shown to be effectively replaced by the culture (pathogen)-based treatments (Vasquez et al., 2017).

In 2017, the full implementation of Guidance For Industry #209 and #213, and the changes to the Veterinary Feed Directive, moved to eliminate the use of medically important antibiotics (i.e., antibiotics important for human medicine) for production purposes (feed efficiency and growth promotion) while still allowing their administration for prevention, control, and treatment of diseases under the supervision of licensed veterinarians (FDA, 2012, 2013, 2018; Bulut and Ivanek, 2022). Although this has already affected the use of medically important antibiotics by conventional farmers (e.g., as evident by the considerable reduction in sales of medically important antimicrobials in the United States in 2017 and 2018; Bulut and Ivanek, 2022), there are still challenges to address to promote further reduction. For example, farmers have a strong personal and financial commitment to the welfare of their animals, which gives workers on conventional dairy farms an incentive to overuse antibiotics due to the purported benefits (Landers et al., 2012; Wemette et al., 2020). Although farmers prefer non-antibiotic disease prevention measures (Wemette et al., 2020), such as better herd management, the monetary and labor investment required presents a considerable cost that may exceed the perceived benefits.

Farmers’ primary concern with respect to antibiotic use is not overuse but consumer preferences, which have moved increasingly away from milk produced by cows on conventional farms, where antibiotics are used (Harwood and Drake, 2018). A new USDA label, “Certified Responsible Antibiotic Use” (**CRAU**), which is currently only in use by the poultry industry for institutional buyers (i.e., unavailable for purchase by the public), could provide these farmers with an incentive to reduce their antibiotic footprint (ARAC, 2018). The CRAU standards differentiate the “therapeutic” and “non-therapeutic” use of medically important antibiotics, and permit only their therapeutic use. The Certified Responsible Antibiotic Use defines “non-therapeutic use” as the administration of medically important antibiotics for growth promotion, feed efficiency, weight gain, and disease prevention, whereas “prevention” is defined as the use of antibiotics in the absence of bacterial disease or exposure to disease as documented by a veterinarian through testing or other means that a pathogenic organism is present in the flock or barn. As part of the CRAU standards for “therapeutic” use, farmers are allowed to use medically important antibiotics only when prescribed by a licensed veterinarian to treat a diagnosed bacterial disease (“treatment”) or to control disease in animals exposed to bacterial infectious agents (“control/metaphylaxis”). Therefore, although the FDA describes “therapeutic use” broadly as the prevention, control, and treatment of disease, CRAU defines the “therapeutic” and “non-therapeutic” use of antibiotics more specifically, and prohibits prevention uses of medically important antibiotics (ARAC, 2018). Thus, CRAU standards restrict antibiotic use more than Veterinary Feed Directive. Farmers’ practices must be certified as meeting CRAU standards, which may require a behavioral change among some farmers. A consumer-facing label touting responsible use of antibiotics, akin to the CRAU certification in poultry, could encourage judicious use of medically important antibiotics among conventional dairy farmers if it increases demand or willingness to pay (**WTP**) for fluid milk produced in this way. Understanding if there is a market for reducing the use of antibiotics in the United States conventional dairy farming is important. Milk produced in such a way would likely carry a smaller premium requirement compared with organic milk (which must be produced by animals raised without antibiotics), but a larger premium compared with that for conventional milk (because reduced antibiotic use in animals is expected to increase the cost of production; The Pew Charitable Trusts, 2015; Ferry, 2019).

The objective of this study was to investigate consumers’ preference for a hypothetical responsible antibiotic use (**RAU**) labeled milk (defined at the start of the Materials and Methods section). The name of the RAU label was inspired by the existing CRAU label. Because this type of labeled milk does not exist on the market, there are 2 important components of consumers’ preferences that must be established. First, to understand the size of the potential market, we must ask consumers whether they would purchase RAU-labeled milk if it were available. Second, if they indicated a willingness to buy this new label, it is important to establish how large a premium, if any, consumers would be willing to pay beyond the price of unlabeled (conventional) milk. Willingness to buy is defined as the likelihood that a consumer intends to purchase a product (Teo and Yeong, 2003), whereas WTP is the maximum amount the consumer is willing to pay for the product (Sellers-Rubio and Nicolau-Gonzalbez, 2016). We investigated the 2 stated questions through a complementary use of 2 exploratory studies. To address the first question, we conducted a nationally representative survey and we employed an experimental Becker-Degroot-Marschak (**BDM**) auction with real milk and money to answer the second question. We hypothesized that in the United States, a market exists for RAU-labeled milk and that consumers would be willing to pay a premium for such milk.

## MATERIALS AND METHODS

For the purpose of this study, the RAU-labeled milk was envisioned to be produced in a way that would minimize antibiotic use more than conventional milk, such as through targeted treatment of mastitis based on a diagnosed causative agent rather than blanket treatment, as is the current practice (Hommels et al., 2021). At the same time, the new label would provide farmers a less coercive incentive to minimize antibiotic use compared with the organic label without jeopardizing animal welfare and thus was envisioned to maximize animal welfare more than organic milk.

### Survey Study

To investigate US consumers’ willingness to buy the hypothetical RAU-labeled milk, we collaborated with the Survey Research Institute of Cornell University, by contributing questions about the new milk label in their 2019 Cornell National Social Survey (**CNSS**). The Survey Research Institute conducts the survey annually by telephone to enroll a random sample of 1,000 participants. Eligibility to participate in the survey is limited to adults, defined as individuals 18 years of age or older, residing in the continental United States. The 2019 CNSS was conducted from August 28 to December 11, 2019, with the approval of Cornell University’s Institutional Review Board (approval #1402004459). The Marketing Systems Group of Horsham, Pennsylvania provided the phone sample, a random digit dial list drawn from the continental United States. The dialed numbers included listed households, unlisted households, and cell phones, but excluded business telephone numbers, disconnected numbers, and non-household numbers. Once phone contact with a randomly selected household was established, the member of the household with the most recent birthday was invited to participate. This sample selection procedure ensures that every household with a phone has an equal chance of being contacted, and once contacted, every adult in the household has an equal chance of participating in the study. The survey was conducted in English using computer-assisted telephone interviewing software. Immediately before the actual survey, the Survey Research Institute conducted a pilot survey with 25 participants identified using the approach identical to that described for the actual survey. Participant feedback on questions contributed by our research team was used to improve question clarity and response quality. Additional information about sample collection is provided in our Supplemental Material (https://doi.org/10.7298/eh5g-yf61; Schell et al., 2022).

### Survey Questions

Through the 2019 CNSS, we collected information on 18 sociodemographic characteristics as well as responses to 2 questions (**Q1**, **Q2**) contributed by our research team (Table 1). The first of the 2 questions, Q1, asked consumers to self-rate their awareness of antibiotic resistance related issues in 2 different ways by randomly asking participants (a) “please rate your level of awareness about the rise of antibiotic-resistant infections” or (b) “please rate your level of awareness about the decline in effective antibiotics,” to assess if the wording and framing of the question affects the response (Table 1). The responses did not differ statistically between the 2 question wordings (chi-squared test: *P* = 0.1). Thus, both versions of this question were combined in subsequent statistical analyses as a variable on “self-rated awareness of antibiotic resistance related issues.” The second of our 2 questions, Q2, asked about willingness to purchase RAU-labeled milk as follows: “there may soon be a new option in buying dairy milk. This option labels milk produced in a way that minimized antibiotic use more than conventional milk and maximizes animal welfare more than organic milk. Would you be willing to purchase this type of milk?” The considered demographic variables from the survey provided information on age, gender, education level, household income, census region, employment status, whether the participant was born in the United States, home ownership status, race, being Hispanic/Latino, number of people (children and adults) in the household, number of children in the household, marital status, political party affiliation, social ideology, religious practice, frequency of attendance at religious services, and whether the survey was taken via landline or cell phone. All demographic questions and response options are shown in Table 2.

### Statistical Analysis of Survey Responses

Demographic characteristics of the survey participants were compared with the US Census data estimated by the US Census and Bureau of Labor Statistics with a one-sample z test (SRI, 2018). The demographic characteristics included in these comparisons were age, gender, education level, household income, employment status, race, and being Hispanic/Latino.

Results of descriptive analyses of Q1 and Q2 and demographic questions are shown in Tables 1 and 2. The self-reported willingness to buy RAU-labeled milk, derived from Q2, was defined as the outcome of interest in univariable and multivariable logistic regression. We employed univariable logistic regression models to screen potential predictors (from among Q1 and demographic questions) for association with the outcome of interest. We converted Q1 into a binary variable with levels (1) “not at all aware” (baseline) and (2) “any other level of awareness” (i.e., “somewhat aware” or “very aware”); furthermore, a single “do not know” response was omitted from analysis and no participants refused to respond. Because our main interest was in comparing consumers’ perceptions regarding unlabeled (conventional) fluid milk and the RAU-labeled fluid milk, the following 2 corresponding response levels in Q2 were used as a binary outcome variable (Table 1): “no, I would continue purchasing conventional milk” (baseline) and “yes, I would purchase such milk” (i.e., the RAU-labeled milk). The remaining responses in Q2 were omitted from further statistical analyses. Statistically significant (*P* < 0.05) variables in univariable analyses were considered as potential predictor variables in the following multivariable logistic regression model:

$$\log\left(\frac{\pi }{1-\pi }\right)=logit(\pi )=\alpha_{0}-\alpha_{1}X_{1}+\alpha_{2}X_{2}+\ldots +\alpha_{k}X_{k},$$

where π=probability of being willing to purchase RAU milk compared with conventional milk; $X_{1}$ through $X_{k}$ are independent variables significant at the univariable level; and $\alpha_{0}$ through $\alpha_{k}$ are the corresponding regression coefficients.

The backward stepwise model selection procedure included a likelihood ratio test, which compared the fit between nested models and indicated whether a given predictor should be included in the final model based on the significance threshold (*P* < 0.05). Akaike information criterion, the Hosmer and Lemeshow goodness-of-fit statistic, and receiver-operating characteristic curves were used to determine whether the final model was a good fit to the data. Odds ratio and the associated profile likelihood 95% confidence interval measured the influence of a variable in the univariable logistic regressions, and the final multivariable logistic regression model constructed for the outcome. The final model was evaluated for the presence of confounding variables to assess whether a non-intervening variable would result in a 20% or greater change in odds ratio between a variable and the outcome of interest, however, no confounding variables were identified. We found no interaction among variables either. R Studio version 1.2.5019 was used for analysis of survey data.

### Experimental Auction Study

Given a new RAU-labeled milk on the market, many consumers may decide they would rather pay a small premium for fewer antibiotics (i.e., pay slightly more for RAU milk compared with conventional milk) than the substantial premiums demanded by organic milk (where no antibiotic are used), which means this new choice could increase consumer welfare. This proposed change in a consumer’s utility function assuming perfect information is subtle, with the model outlined below:

$$\begin{array}{l} U_{i}=\alpha_{0i}+\alpha_{1}Price_{i}+\alpha_{2}Organic_{i}\\ +\sum_{k=1}^{2}\alpha_{3}^{k}AntibioticUse_{k,i}+X\beta_{i}, \end{array}$$

where $U_{i}$ is the utility a person gets from the purchase of fluid milk, i is an indicator for individual i, k is an indicator for whether or not antibiotics are used, *Price* is the price of a half-gallon of milk, *Organic* is a dummy variable on whether the product is organic, *Antibiotic Use* represents 2 levels (responsible use and no restriction on antibiotic use), X is a vector representing the attributes of milk abstracted away from in this study, including brand and fat content, β is a vector of coefficients corresponding to the X vector of milk attributes abstracted away from. As is convention, α represents marginal utilities for each attribute. If the RAU label truly provides value to consumers, we would expect the marginal utilities derived from both conventional and organic milk to decrease for at least some consumers from the new label’s introduction.

To determine the relative WTP for different milk labels, we conducted a BDM experimental auction study on August 7, 2019, approved by Cornell University’s Institutional Review Board (approval #1906008889). The experimental auction with milk (as well as a practice auction with bottled water) took place in Cornell’s Lab for Experimental Economics & Decision Research (**LEEDR**) in Ithaca, New York. Experiment subjects were recruited via announcements sent through LEEDR e-mail list servers, which include Cornell students, Cornell staff, alumni of the Charles H. Dyson School of Applied Economics and Management, and Ithaca community members who signed up to participate in experiments at LEEDR. Any participant older than 18 years of age who does not suffer from lactose intolerance was eligible to participate in this experiment. Three experiment sessions were conducted, and a total of 85 participants were recruited. A power-based sample size calculation could not be conducted because we had no WTP data on the RAU milk label. Each experiment session lasted approximately 1 h, with 15 min for informed consent, and 45 min to conduct experiment, including 15 min to distribute educational materials and explain the nature of the auction. Each participant received $35, part of which they used to participate in the experimental auction. At the end of the experiment, each participant answered a short demographic questionnaire (Supplemental Material; https://doi.org/10.7298/eh5g-yf61; Schell et al., 2022); moreover, participants’ characteristics were compared with the US Census data estimated by the US Census and Bureau of Labor Statistics with a one-sample z test (SRI, 2018).

In a BDM auction, participants first place sealed bids on the products presented. After receiving the bids, the researcher draws a random number from a set range. If the participant’s bid equals or exceeds this drawn number, they purchase the product for the price drawn. If the participant’s bid is lower than the number drawn, no exchange occurs. We worried about the potential biasing effect of providing participants with a price range within which we randomly drew the price. However, we decided to draw a random number between $0 and $6, which covers the most extreme retail prices customers could see at a supermarket and, given the familiarity of milk purchasing decisions, people should already have adequate information to make a reasonable bid.

To encourage realistic estimates, we randomly selected 1 round of the auction as binding and then draw a random price against which the subject’s bid is compared and, if the bid is higher, they pay the random price. This method is consistent with economic theory; that is, research has established consumers make purchases subject to budget constraints, and they value products based on the sum of their attributes (Tanrikulu, 2021). These practices correspond to the Lancaster’s Characteristics Theory of Value (which posits that all goods possess characteristics or attributes that are demanded by the consumers, not the goods themselves; Alfnes et al., 2006). The BDM should also be demand-revealing, as participants must balance their enjoyment of a product with the probability that their bid wins them this product and they must pay for it (Becker et al., 1964). We only conducted 3 rounds of auctions with the milk, lower than many previous studies, based on findings in a recent study on WTP for genetically modified apples, which revealed that consumers’ feelings of boredom affected their bidding behavior as the number of rounds increased (Kassardjian et al., 2005). If participants find the decision-making process arduous or tedious, they would incur a transaction cost to making decisions, which violates one of the chief assumptions ensuring the external validity of experimental auctions (Smith, 1976).

**Figure 1.**
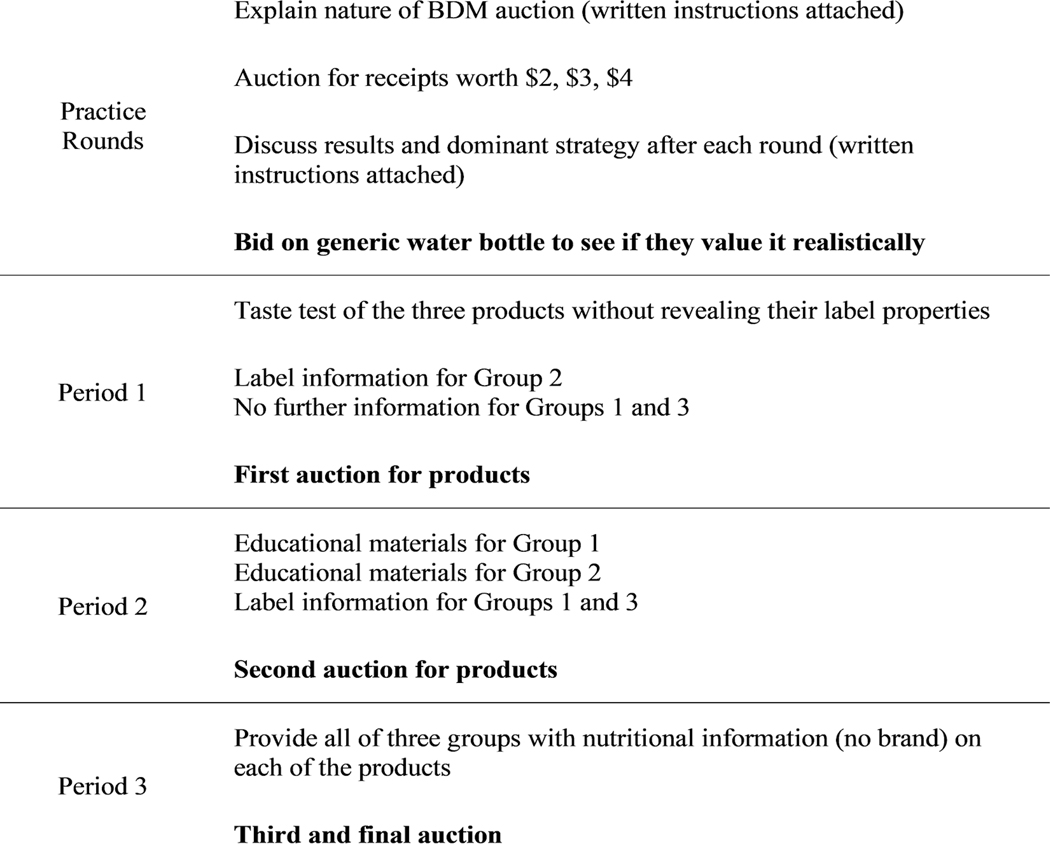
Sequence of events for participants in the experimental Becker-Degroot-Marschak (BDM) auction, each randomly allocated to 1 of the 3 treatment arms (groups).

Because of the unfamiliar setting of an experimental auction, untrained participants could produce inaccurate WTP bids (Corrigan and Rousu, 2008). In fact, in the original paper on the BDM auction, the authors found that repeated sessions made people act more in accordance with how they should, based on the optimal strategy (Becker et al., 1964). Training allows for learning and drives home the fact that the money and consequence of the bid are real. If participants learn to play the game intelligently in advance, we should not have to worry about the effect of learning biasing the results during the actual experiment. Therefore, we decided to conduct several induced value practice rounds (Figure 1), where participants bid on monetary values and see their results compared with the optimal payoff, followed by an explanation of the optimal strategy and a bid on a water bottle, a product with relatively little product differentiation, to determine whether participants’ knowledge of the bidding process translated to their valuation of real products. The water bottle auction was non-hypothetical to show participants how they win the auction. After establishing the optimal strategy through practice rounds, the milk auction began.

**Figure 2.**
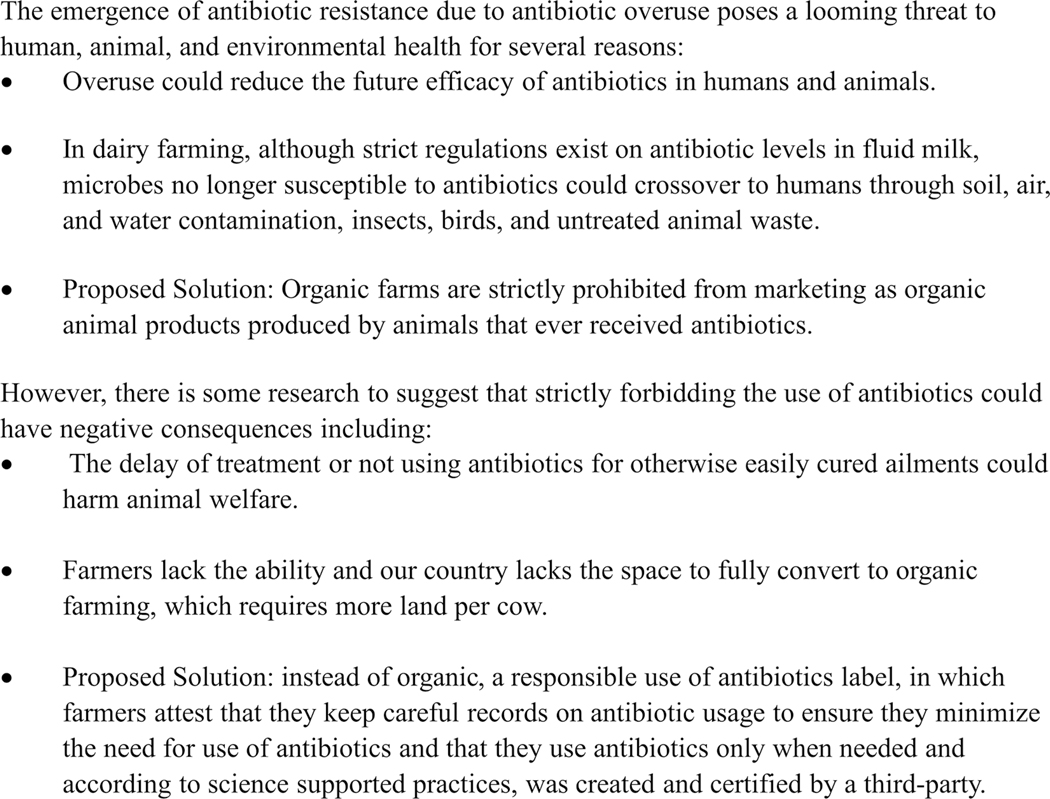
Educational materials on antibiotic use in the experimental auction.

We conducted a double blind 3-arm trial where participants and administrators were blinded to treatment. Computers in the LEEDR laboratory randomly allocated participants into 3 treatment groups (Figure 1). All 3 groups taste tested the 3 products without revealing their label properties. Group 1 first blindly bid on the 3 milk options and then received both an educational treatment (depicted in Figure 2) on antibiotic use in the production of milk and were shown the labels of each type of milk between the first and second round of bidding. Group 2 received the label reveal before the first round of bidding, but the educational treatment between the first and second round of bidding. Group 3 (control) received the label reveal between the first and second round of bidding, but they never receive the educational treatment. This constitutes both a between- and within-subjects design, where the results of an individual participant’s bidding before and after education and the results of groups with different treatments can be compared. This study should reveal consumers’ true WTP for organic labeled milk, the proposed RAU-labeled milk, and unlabeled milk (Becker et al., 1964). The decision to use a non-hypothetical experimental auction lies in our hope to avoid social desirability bias in stated preference experiments (Noussair et al., 2004; Van Loo et al., 2011; Quaife et al., 2018). We informed participants they could take the milk home, have us store it for the day, or have us deliver it to them to prevent bias from the inconvenience of handling a perishable product.

Because the RAU label does not exist on the market, we had to substitute organic milk during the actual exchange of goods. This hypothetical label would include organic milk, so we decided organic milk served as an appropriate substitute. We explained this and why we had to do it to subjects who won the RAU-labeled milk at the end of the experiment before any exchange occurred. The general process of the experiment for each arm is detailed in Figure 1.

### Tobit Model Specification

We conducted between-group comparisons of treatment effects to determine their generalizability and whether we can pool the sample. A maximum likelihood estimation technique is used to compare the asymptotic means between groups given identical treatments. Specifically, we test whether the blind bidding results in groups 1 and 3 differ, compare the effect of label information on bidding in groups 1 and 2, and the effect of the education treatment in groups 2 and 3. This process reveals whether the timing of the education treatment and label information causes changes in bids due to ordering effects. Given no statistically significant differences, we pool the data between groups by treatment to increase the sample under analysis and gain an understanding of the effect of education and labeling on bidding behavior. We next explore within-subject comparisons to control for time-invariant fixed effects and determine how bids changed for participants as the information they received changed over the course of the study.

To determine the effect of different treatments and demographic characteristics on a participant’s WTP for milk products, we used a 1-limit mixed effects Tobit model. Although we forced consumers to provide bids between $0 and $6, which could cause lower and upper bound censoring, none of the bids came close to $6. As a result, there was no need to control for right censored data and a 1-limit model will suffice. However, economic theory dictates that all $0 bids should not be perceived as equal. For instance, 1 participant who bids $0 may still derive less utility from a milk product than another $0 bidder. Because one cannot bid a negative value, some consumers cannot bid their true value. Thus, we use a Tobit model because it implies the existence of an unmeasurable latent variable, in this case how much a consumer truly wishes to bid, that we want to analyze. Therefore, the following piecewise function shows how the value of the true bid varies depending on whether it falls in the prescribed range of possible bids:

$$bid_{ij}^{*}=\{\begin{array}{c} 0\;\text{if}\;bid_{ij}^{*}\leq 0\\ bid_{ij}^{*}=X\beta +Z\tau +\varepsilon_{i}\;\text{if}\;bid_{ij}^{*}\geq 0\;\text{and}\;\leq 6 \end{array},$$

where $X\beta +Z+\varepsilon_{i}$ is

$$\begin{array}{l} bid_{Milk}=\beta_{0}+\beta_{1}NoInfo_{i}+\beta_{2}EducTmt_{i}\\ +\beta_{3}NutInfo_{i}+\sum_{k=1}^{3}\alpha_{1}^{k}TmtGroup_{i}\\ +\left(\begin{array}{c} \beta_{1}NoInfo_{i}+\beta_{2}EducTmt_{i}\\ +\beta_{3}NutInfo_{i}+\sum_{k=1}^{3}\alpha_{1}^{k}TmtGroup_{i} \end{array}\right)\beta_{4}Organic_{i}\\ +\left(\begin{array}{c} \beta_{1}NoInfo_{i}+\beta_{2}EducTmt_{i}\\ +\beta_{3}NutInfo_{i}+\sum_{k=1}^{3}\alpha_{1}^{k}TmtGroup \end{array}\right)\beta_{5}RespAntibioticUse\\ +Z_{i}+\varepsilon_{i}, \end{array}$$

with

$$\varepsilon_{i}∼N\left(0,\sigma^{2}\right).$$

The variable $Z_{i}$ represents a vector of the demographic, taste, and belief variables that affect an individual’s bid, outlined in Supplemental Table S1 (https://doi.org/10.7298/eh5g-yf61; Schell et al., 2022), and $\varepsilon_{i}$ is an independent and identically normally distributed error term. *NoInfo* is the effect of having no additional information about a product, *NutInfo* is the effect of having nutritional facts about a product, and *EducTmt* is the effect of receiving education about the meaning of RAU. i is for individual i, j is for choice j, and k is an indicator for the individual being in 1 of the 3 treatment groups. β in Xβ represents the impact of treatment group, milk label, and the treatments received. We created dummy variables for each possible treatment (blind, educational materials, and nutrition information) and interacted them with organic and RAU-labeled milk dummy variables (*Organic* and *RespAntibioticUse*, respectively), with the label information rounds and unlabeled milk acting as the reference categories. We expect heteroskedasticity in the model due to the relatively small and demographically specific group of people who have historically paid large premiums for organic milk, so the model uses Huber-White robust standard errors. Last, we check for multicollinearity because consumers often perceive health, antibiotic use, and environmental consciousness as going hand-in-hand for food products, which means these survey questions could explain the same variation in bidding behavior (Gil et al., 2000; Lusk and Briggeman, 2009; Van Loo et al., 2011; Costanigro et al., 2012). After extracting the correlation matrix, we found the variance inflation factors suggest little evidence of multicollinearity.

We derive our hypotheses regarding the effect of each demographic variable in Supplemental Table S1 (https://doi.org/10.7298/eh5g-yf61; Schell et al., 2022) from a review of the literature. Age should have a parabolic effect on bids for RAU and organic milk, as young adults tend to spend the most for organic milk, although older Americans generally have more negative views of antibiotic use reflected in their WTP (Nayga, 1996; Bernard and Mathios, 2005; Van Loo et al., 2011). Those with children tend to have greater concerns regarding antibiotics (Wun et al., 2012) but are willing to pay lower premiums for organic milk, likely due to budget constraints (Schrock, 2010). As a result, we created an interaction term between household income and children under 18 years old to determine the budget per child. This change means more children under 18 years old (holding the budget per child constant) should increase WTP for reduced or no antibiotic use in fluid milk production. Women, primary shoppers, Black and Hispanic respondents, respondents with more education, and respondents with higher incomes are all more likely to pay more for labeled milks because of their history of worrying more about antibiotic use in food production (Nayga, 1996; Harwood and Drake, 2018; Gundala and Singh, 2021).

Frequency of milk and size of milk purchased should decrease the premium paid for labeled milks because these habits would take a greater toll on a participant’s weekly budget. Higher self-rated knowledge of antibiotics, concern for the environment, and concern for one’s health should increase the premiums for labeled milks because they demonstrate a consumer who cares more about the issues these milk varieties seek to address. We do not know how a participant’s preferred method of milk consumption or fat content typically purchased will affect bidding behavior, but included these variables given their importance in driving overall demand for milk (Kim et al., 2013; Bir et al., 2019). R Studio version 1.2.1 was used for analysis of the experimental auction data.

## RESULTS

### Survey Study

Among the 1,000 participants in the 2019 CNSS, the average age was 47 years, 893 (89.4%) were born in the United States, 793 (79.7%) self-identified as Caucasian, 713 (71.9%) were religious, 692 (71.6%) reported an annual household income of over $50,000 and 618 (61.9%) were employed (Table 2). The questions with the highest rates of refusals were political party (n = 20), household income (n = 18), and social ideology (n = 17).

Age (*P* = 0.9), gender (*P* = 0.9), race (*P* = 0.1), and household income (*P* = 0.1) compositions in the 2019 CNSS were not significantly different from those reported in the US Census. However, fewer survey participants described themselves as being Hispanic/Latino compared with the US Census (*P* = 0.005, 10% participants being Hispanic/Latino versus 17% identified in the US Census). In addition, the employment rate among survey respondents was significantly higher (62%) than the US Census (58% employment in the US; *P* = 0.017) and they had higher education (*P* = 2.5e-7; 46% of the participants had a bachelor’s degree or higher in the survey versus 28% in the US Census).

Most survey participants (75.1%) described themselves as aware of antibiotic resistance related issues (Q1, Table 1). About 20.8% (207/994) of the participants reported that they do not purchase dairy milk (Q2). When asked about their willingness to buy organic, conventional, or RAU-labeled milk, almost half of the respondents (48.5%; 482/994) responded that they would buy a RAU-labeled milk. On the other hand, about 30.7% (305/994) of the survey participants reported that they would continue buying organic (8.4%) or conventionally (22.3%) produced milk. These results suggest enormous potential demand for the new RAU label, with an implied market share superior to either of the existing milk options. Although it does not provide direct information on WTP, the large number of converts from organic to the RAU label would suggest the possibility of a significant premium.

Univariable analyses revealed 12 out of 18 demographic variables serve as possible predictors of the difference between the consumers who would purchase RAU-labeled milk, compared with those who would continue purchasing conventional milk (Table 3).

Multivariable logistic regression identified self-rated awareness of antibiotic issues, as well as age, education level, and social ideology as the best predictors of preference for RAU milk compared with preference for conventional milk. Respondents who would purchase RAU milk compared with conventionally produced milk were more likely to describe themselves as younger, aware of issues with antibiotic resistance, moderate (social ideology), and were better educated (Table 4).

The final logistic regression model describing purchasing preference between RAU milk and conventionally produced milk had an Akaike information criterion of 782.45 and a residual deviance of 768.45. The null model had an Akaike information criterion of 859.69 and a residual deviance of 857.69. A likelihood ratio test revealed that the fit of these models was significantly different (*P* < 2.2e-16). Construction of receiver-operating characteristic curves (AUC = 0.80) and Hosmer-Lemeshow test (40 bins/intervals, χ^2^ = 35.8, df = 38, *P*-value = 0.5) suggested a good fit for the finalized multivariable logistic regression model, which implies that the preference for RAU label over unlabeled milk, at least in the hypothetical survey scenario, reflects a practically and statistically significant effect.

### Experimental Auction

Because the sample consisted of residents of Ithaca, New York, it is not surprising that the experimental auction participants are not generalizable to the broader American public. On average, subjects were older (*P* < 2.2e-16; 61% of the auction participants were older than 35 years old, whereas the same population comprises 38% of the US population; US Census Bureau, 2020), had different racial composition (*P* = 0.011; white: 69/85 = 81% in our study, 76% in the US population; black: 2/85 = 0.02% in our study, 14% in the US population; US Census Bureau, 2019), and had higher levels of education [*P* < 2.2e-16; 69% of the sample had bachelor’s degree or higher compared with the US population (35%; US Census Bureau, 2021)] and income (*P* = 3.1e-14; 77% of the participants had household income higher than $50,000, which is 65% in the United States; US Census Bureau, 2020). They self-reported an above average environmental (3.87 out of 5) and health consciousness (3.42 out of 5; Supplemental Table S1; https://doi.org/10.7298/eh5g-yf61; Schell et al., 2022). Subjects were also disproportionately female (*P* = 2.3e-11; 78% of the auction participants were female, whereas the United States has 51% female population; US Census Bureau, 2020) and the primary shopper in the household, which is an advantage given how much consumption decisions are still driven by these 2 groups. Table 5 shows the covariates between the 3 treatment arms were well balanced. Despite its low external validity, this study could prove vital in determining the viability of a RAU label because it represents a high income, female, and relatively young demographic that, at least by the theories outlined in the existing literature, should be more willing to pay for RAU than the average consumer (Nayga, 1996; Bernard and Mathios, 2005). In the same way that organic milk’s premium is driven largely by demographic outliers who will pay significantly more for the product, those willing to pay a premium for RAU may exist chiefly in this young, female, high-income demographic.

Table 6 pools treatment arms by the amount of information available at the time of the bids for each group regardless of bidding round. Although the differences are not statistically significant, there appears to be a “general aversion to RAU labeling in the absence of further education.” This striking result suggests even among this sample of respondents, who consider themselves to have an average- to above-average understanding of antibiotics, the use of antibiotics in conventional dairy farming remains relatively poorly understood. The negative reaction may be a consequence of unintentional signaling about safety (Abrams et al., 2010) by even just mentioning the word “antibiotic” in the RAU label because consumers view antibiotics as unnatural, unnecessary, or harmful (Schwendel et al., 2015; Clark et al., 2016; Singer et al., 2019). After receiving information on what each label entails, the negative effect of the responsible use label dissipates. However, participants do not exhibit a WTP a premium for RAU-labeled milk compared with unlabeled milk, a trend which is in sharp contrast to the persistent premium participants report being willing to pay for organic milk in the literature.

Table 6 represents both the estimated WTP given only label information with no education about the new label and the estimated WTP when all consumers are educated about the label. However, a more realistic market scenario would be with some proportion of consumers having knowledge of the label and all having access to nutrition information. To simulate these conditions, Table 7 displays the WTP for groups 1 and 2, who receive education, labels, and nutrition information, combined with the control group (group 3), who only receive labels and nutrition information. We cannot preclude the possibility of unmeasured confounding between these groups; however, the balance on covariates shown earlier suggests that the education treatment, or its absence, should theoretically affect all 3 groups similarly. These results suggest that there could be more promise in the RAU label than Table 6 revealed.

The regression shown in Supplemental Tables S2 through S4 (https://doi.org/10.7298/eh5g-yf61; Schell et al., 2022) reveals which covariates had a statistically significant effect on WTP for each of the 3 milk products. In the blind bidding rounds for each product, level of education had a strong negative effect on WTP, whereas an increase in the number of children in a household, status as a primary shopper, and health consciousness all had a positive effect on the reported WTP. This finding corresponds well with the literature on the largest consumers of milk, who tend to have children, lower levels of education, and value its potential health benefits (Davis et al., 2012). After revealing the labels, better-educated subjects continue to exhibit a lower WTP for all 3 milk products, whereas status as the primary shopper had a positive effect on WTP for unlabeled and organic milk and, counterintuitively, greater health consciousness also resulted in an increase in WTP for unlabeled milk. The lack of positive covariate associations with WTP for RAU-labeled milk could suggest that the significant and nearly universal aversion to antibiotic use in agriculture causes a negative effect on demand for a product that even references antibiotic usage (Wemette et al., 2021).

In the rounds during which subjects receive a brief educational excerpt detailing the meaning of the labels, the number of children in a household has a significant and positive effect on WTP for unlabeled and organic milk. Frequency of milk purchase exerts a significant and positive effect on WTP for unlabeled milk. Finally, health consciousness increases WTP for organic milk. These 3 findings align with the previous literature, which found both that households with more children, which typically also buy more milk via frequency or quantity, had a greater preference for unlabeled milk (Davis, et al., 2012), and that health consciousness is a key driver of the consumption of organic milk (Messer et al., 2017). Even after the label reveal, the only significant covariate in the demand for RAU-labeled milk remains the negative effect of higher levels of education on WTP (Supplemental Table S3; https://doi.org/10.7298/eh5g-yf61; Schell et al., 2022). This could once again point to the general negative perception of antibiotic use in agriculture, although why higher education levels remain so important in driving down WTP is unclear.

Last, after incorporating nutritional information about each milk product, the number of children in a household and health consciousness were found to be strong and positive predictors of WTP for organic and unlabeled milk. However, none of the demographic variables had a significant effect on WTP for RAU-labeled milk. This result could suggest that the educational materials combined with nutritional information have eliminated the variation in demand for RAU-labeled milk for people with different demographic characteristics in our experimental auction, although the reasons for the lack of differences remains unclear.

## DISCUSSION

In this study, we considered the potential market for and price of a hypothetical RAU-labeled fluid milk product. The RAU-labeled milk was described to consumers as an intermediate option between the 2 current modes of production systems regarding antibiotic use (conventional and organic). To establish the existence of a potential market for this label and consumer WTP, we used 2 complementary methodologies, a survey, and an experimental auction. The main findings from each approach and their limitations are discussed below.

About half of the participants in the 2019 CNSS reported that they would purchase the new RAU label, described to them as milk produced in a way that minimizes antibiotic use more than conventional milk and maximizes animal welfare more than organic milk. This finding indicates an affinity among a considerable portion of the US dairy milk consumers for the RAU milk, and thus suggests that there may be a market for this new label. This level of consumer interest would be of great value for promoting judicious use of antibiotics in the dairy industry, considering that consumers are increasingly concerned regarding the overuse of antibiotics in animal agriculture and animal welfare (Boogaard et al., 2008; Miele, 2010; Barlow, 2011; You et al., 2014; Cardoso et al., 2016; Clark et al., 2016; Pieper et al., 2016; Cardoso et al., 2019). Importantly, it has been reported that consumers who expressed concerns about dairy cattle welfare also supported avoiding antibiotic use in dairy production (Wolf et al., 2016). They believe that improved animal welfare due to reduced antibiotic use would ensure healthier and better products (Kanter et al., 2009), although their concerns about antibiotics reflect fears about residues rather than resistant bacteria ending up in the milk supply (Gil et al., 2000; Harwood and Drake, 2018; Wemette et al., 2020).

Our findings did indicate there was a market for RAU-labeled milk, though it was a specific one. In our survey, respondents who would prefer RAU milk over conventionally produced milk described themselves as younger, aware of issues with antibiotic use, politically moderate, and having higher education. According to the literature, people who value organic milk are identified as generally female, higher income, younger, and have no children, with a far higher WTP than their peers (Bernard and Mathios, 2005; Van Loo et al., 2011; Clark et al., 2016; Bulut et al., 2021). However, organic milk buyers and those willing to pay more for better animal welfare differ markedly from the demographics most concerned about antibiotic use in agriculture. Consumers most concerned about antibiotic use in agriculture are also female as the prior group, but they tend to be nonwhite, live in metropolitan areas, and are older and less educated (Nayga, 1996; Bernard and Mathios, 2005). As a result, there are several distinct demographics to whom this new label could hold great appeal; specifically, those who prefer the animal welfare component and those who prefer the promise of reduced use of antibiotics. The new label’s claims about animal welfare and antibiotics may explain why such a large portion of consumers indicate they would purchase RAU milk if available.

We do not know whether consumers oppose antibiotic use in milk in principle or if they oppose perceived overuse on conventional farms. Some key aspects of opinions are that they tend to be formed by simple heuristics that may even violate basic logic rules (Tversky and Kahneman, 1983), and misinformed opinions are frequently associated with overconfidence (Radecki and Jaccard, 1995). However, some evidence suggests that people will compromise on an attribute they dislike, such as antibiotic usage, in exchange for lower prices. For instance, a study of perceptions of genetically modified organism in food found a WTP more for and accept products with low, but nonzero, genetically modified organism thresholds compared with products with no threshold (Noussair et al., 2004). Similarly, another study found people with young children expressed strong concerns about antibiotic use and growth hormones that diverge with their general unwillingness to buy organic milk (Hwang et al., 2005), which likely stems from the cost of organic milk relative to consumption needs and budget (Bir et al., 2019; Ortez et al., 2021). This result suggests that despite consumers’ aversion to antibiotic use in milk production, a market could exist for simply reducing their administration in exchange for a price premium lower than for organic milk.

The results of the auction experiment conducted in this study suggest that consumers first responded negatively to the new RAU label. However, after receiving educational treatments explaining the effect of excess antibiotic use on animal, environmental, and human welfare, consumers exhibited a slight, statistically insignificant preference for RAU-labeled milk over unlabeled milk. The 2 groups that received nutrition, education, and label information exhibited a premium of $0.06 to $0.08 for the RAU label (for half-gallon of milk). Although these results are statistically insignificant, the difference in the point estimates suggests that a premium that may not be too different from the one enjoyed by recombinant bST-free milk ($0.26 per half-gallon of milk in 2001; Bernard and Mathios, 2005) could exist for RAU-labeled milk. This may provide an incentive for farmers to cohere to judicious antibiotic use standards. Importantly, milk production under the proposed RAU label would likely require increased labor or capital costs due to changes associated with the animal production system (USDA ERS, 2016b), including additional preventive measures on the farm such as biosecurity measures (e.g., on-farm sanitation, vaccination, bacterial testing, and so on; Lhermie et al., 2020).

The current study is the first step in understanding viability of the new RAU milk label as it aims to generate a preliminary understanding of its marketability. The next step in assessing the viability of this new label would be assessing whether a slight increase in consumer WTP for the RAU label would justify the expected increased production costs incurred by farmers transitioning from the production of unlabeled milk. Should a transition be economically viable, such a finding could prove important both for human and animal welfare and in revitalizing the declining non-organic dairy industry. Thus, further research is needed to determine whether the savings from reduced antibiotic use and any increase in sale or marketing potential of the new milk label would offset or hopefully surpass the potential increase in costs.

The premium for organic milk in our auction study was much higher than for the new label (mean WTP of $2.24 for organic milk versus $1.92 for RAU milk per half-gallon) even with the educational intervention, which may be due to the respondents’ general concerns about antibiotics. In an experimental auction conducted by Bernard and Bernard (2009), the authors show that a consumer with a 100% belief that conventional milk contained milk from cows treated with antibiotics would be willing to pay $0.63 more for antibiotics-free milk than a consumer with a 0% belief. It is possible that only the complete elimination of antibiotics will compel consumers to pay this premium.

The survey and experimental auction studies described have several limitations. The use of the 2 complementary methodologies was beneficial because it allowed us to explore different components of consumers’ perceptions about the proposed new milk label using methods used in multiple disciplines. However, the results from the 2 methodologies are not completely comparable, because the study populations were different and the technical differences between the 2 methodologies precluded describing the hypothetical milk product to study participants in exactly the same way in both methods. For example, we cannot be sure whether the survey participants understood the potential animal welfare aspect of prohibited antibiotic use in the production of organic dairy products in the same way this was explained to participants in the experimental auction (Figure 2). Regarding the 2019 CNSS, most prominently, social desirability bias could drive consumers to prefer the label (due to what the label represents to them or the way it was framed) because it is “the right thing to do” and not as a reflection of their actual preferences. Consumers tend to vastly inflate their willingness to buy in hypothetical surveys because this decision is nonbinding, especially when the label represents a “social good,” which results in little external validity (Little and Berrens, 2004; Noussair et al., 2004; Van Loo et al., 2011; Grebitus et al., 2013; Quaife et al., 2018). Our survey sample was aimed to be representative of the US population and it was representative in terms of age, gender, household income, and race. However, compared with the US Census, our survey participants were significantly better educated, had higher level of employment, and included fewer people from the Hispanic/Latino origin, which could have introduced selection bias into the study. There is also the possibility of misreporting in both the survey study and the short demographic questionnaire at the end of the experimental auction. For example, as the survey was self-report, there was no objective external assessment of antibiotic knowledge, so the reliability and validity of findings as they relate to antibiotic knowledge, particularly in connection to milk production, is limited.

There are several important limitations to the experimental auction as well. Because participants’ age, education, and income levels, as well as gender and racial composition, in the trial were different than among adults in the general public (US Census Bureau, 2019, 2020, 2021), the results of the auction are less generalizable than the CNSS. We asked participants about their education level, but not if they were students at the time of the auction; however, considering that 61% of auction participants were older than 35 years old, 69% completed bachelor’s or higher degree, and 77% had household income higher than $50,000, the number of students in the sample was likely relatively low. Prior research indicates a lack of significant differences between the WTP bids of students and non-students in experimental auctions for food goods (Depositario et al., 2009), supporting that our results are representative irrespective of the student status of the auction participants. Additionally, the sample was too small to capture slight differences in WTP between labels, which appears important given the relatively small difference between WTP for RAU-labeled and unlabeled milk. Ultimately, it appears that consumers’ beliefs about their preferences align poorly with their true demand (i.e., they prefer RAU milk but are unwilling to pay), which may be due to social desirability bias known to exist in hypothetical scenarios (Lusk and Norwood, 2006) and self-reporting of health and food-related data (Bir et al., 2020). Furthermore, the powerful effect of the educational intervention in a reportedly “high knowledge” sample suggests that consumers’ self-rated knowledge regarding antibiotic use in milk production overestimates their actual understanding. Subject to these limitations, the value of the present study is in the obtained baseline data about consumers’ preferences for the RAU milk label from the survey. Additionally, the study obtained preliminary estimates of consumers’ WTP for the proposed milk label. As such, the study is expected to aid follow-up investigations of the new RAU milk label as a strategy to reduce antibiotic use in conventional dairy farming in the United States.

## CONCLUSIONS

Numerous existing studies examine how consumers respond to organic labels; however, this study is the first to our knowledge to explore how they respond to labels that focus on the responsible (minimized) use, and not the elimination, of antibiotics in conventional dairy farming. This option, should there be a sustained market and financially viable mode of production, could have transformative implications for the dairy industry and public health. Our survey study showed that about 50% of consumers state an interest in the hypothetical RAU-labeled milk. However, in the experimental auction study participants were not willing to pay a significant premium for the new label compared with unlabeled milk, though a premium for organic milk remained. There is also a possibility that our study was underpowered to detect a significant difference in WTP for RAU and unlabeled milk. Thus, consumers’ preference for the RAU-labeled milk could reflect the social desirability bias, but may also indicate their genuine preference for which they will not pay a substantial premium. Importantly, the hypothetical survey and experimental auction with their own unique strengths and weaknesses demonstrate the importance of using complementary methods to understand the potential market for a new product.

## Figures and Tables

**Table 1.** Responses to survey question 1 and 2 as part of the 2019 Cornell National Social Survey^1^

Item	Number	Percent^2^ (%)

Question 1: Self-rated awareness of antibiotic resistance-related issues—Independent variable		
Please rate your level of awareness about the rise of antibiotic-resistant infections/the decline in effective antibiotics		
Very aware	366	36.6
Somewhat aware	385	38.5
Not at all aware	248	24.8
Do not know	1	—
Question 2: Willingness to purchase responsible antibiotic use milk—Outcome There may soon be a new option in buying dairy milk. This option labels milk produced in a way that minimized antibiotic use more than conventional milk and maximizes animal welfare more than organic milk. Would you be willing to purchase this type of milk?		
No, I do not purchase dairy milk	207	20.8
No, I would continue purchasing conventional milk	222	22.3
No, I would continue purchasing organic milk	83	8.4
Yes, I would purchase such milk	482	48.5
Do not know	6	—

1 Sample size = 1,000.

2 Percentages were calculated after excluding “refused” and “do not know.”

**Table 2.** Sociodemographic variables describing characteristics of the 2019 Cornell National Social Survey respondents^1^

Item	Number^2^	Percent^3^ (%)

Gender		
Male	507	50.8
Female	486	48.7
Other	5	0.5
Refused	2	—
Education level (last grade or class that was completed in school)		
High school (grade 12 or General Educational Development certificate) or less	250	25.0
Technical, trade, or vocational school after high school, or some college, no 4-year degree (including 2-year associate degree)	291	29.1
College graduate (bachelor of science or art, or another 4-year degree)	261	26.1
Postgraduate training or professional schooling after college	197	19.7
Refused	1	—
Household income		
Under $50,000	274	28.4
$50,000 to under $100,000	348	36.0
$100,000 or over	344	35.6
Do not know	16	—
Refused	18	—
Census region (geographic area consisting of several states defined by the US Department of Commerce, Bureau of the Census)		
Northeast	188	18.8
Midwest	232	23.2
South	365	26.5
West	215	21.5
Born in the United States		
No	106	10.6
Yes	893	89.4
Refused	1	—
Employment status (Last week, did you do any work for pay? Include any job from which you were on vacation or otherwise temporarily absent, e.g., “on layoff.”)		
Employed	618	61.9
Unemployed	165	16.5
Retired, disabled, or unable to work	216	21.6
Refused	1	—
Hispanic or Latino (Hispanic origin or descent, such as Mexican, Puerto Rican, Cuban, or some other Spanish background)		
No	898	89.9
Yes	101	10.1
Refused	1	—
Home ownership status		
Own or live there rent free	670	67.1
Rent	328	32.8
Refused	2	—
Race		
White or Caucasian	793	79.7
Black or African American	130	13.1
Native American (i.e., American Indian, Aleut, Eskimo)	52	5.2
Asian or Pacific Islander	55	5.5
Other only	41	4.1
Refused	9	—
People in household		
Adults over age 64, alone or with children under age 18	180	18.0
Adults (age 18–64)	818	82.0
Refused	2	—
Number of children (under age 18) in household		
No	661	66.4
Yes (1 to 7)	334	33.6
Refused	5	—
Marital status		
Married	490	49.5
Single	312	31.5
Divorced, separated, widowed	186	18.8
Other partnership^4^	2	0.2
Refused	10	—
Political party		
Democrat	386	39.4
Independent	208	21.2
Republican	360	36.7
Other party affiliation	26	2.7
Refused	20	—
Social ideology		
Liberal	287	29.2
Moderate	342	34.8
Conservative	354	36.0
Refused	17	—
Religious affiliation		
No religion, atheist, or agnostic	279	28.1
Protestant, Catholic, Christian Orthodox, Jewish, Muslim, or other non-Christian religion	713	71.9
Refused	8	—
Frequency of attendance to religious services		
Once a week or more often	292	29.2
Once a month to a few times a year	301	30.2
Seldom to never	405	40.6
Refused	2	—
Cell phone or traditional landline phone for survey		
Landline	26	2.9
Cell phone or voice over IP (Skype, Vonage, etc.)	968	97.1
Refused	3	—

1 In terms of continuous variables, the median (interquartile range) for age of participants (n = 1,000) was 47.0 (32–61) years.

2 Sample size = 1,000.

3 Percentages were calculated after excluding “refused” and “do not know.”

4 Other partnership includes boyfriend/girlfriend, common-law married, or domestic partnership.

**Table 3.** Characteristics of the respondents^1^ that were different (*P* < 0.05) among those who self-reported intent to purchase the responsible antibiotic use-labeled milk compared with the ones who would instead continue purchasing unlabeled (conventional) milk

Predictor	OR^2^	95% CI^3^

Survey question 1: Self-rated awareness of antibiotic resistance related issues		
Not at all aware	1.00 (ref^4^)	
Any other level of awareness (somewhat aware/very aware)	2.36	1.65–3.36
Demographic variables		
Age (n = 700)	0.97	0.96–0.98
Education level (n = 703)		
High school or less	1.00 (ref)	
Technical trade, vocational school after high school, or some college, or college graduate	1.77	1.23–2.56
Postgraduate training or professional	3.27	2.00–5.49
Household income (n = 681)		
Under $50,000	1.00 (ref)	
$50,000 to under $100,000	1.36	0.91–2.02
$100,000 or over	2.30	1.52–3.50
Census region (n = 704)		
Northeast	1.00 (ref)	
Midwest	0.57	0.34–0.93
South	0.68	0.43–1.08
West	0.99	0.59–1.69
Employment status (n = 703)		
Unemployed, retired, disabled, unable to work	1.00 (ref)	
Employed	1.85	1.33–1.69
Race—Asian or Pacific Islander (n = 702)		
No	1.00 (ref)	
Yes	2.32	1.01–6.25
Marital status (n = 698)		
Single	1.00 (ref)	
Married	0.91	0.62–1.32
Divorced, separated, or widowed	0.48	0.30–0.76
Political party (n = 677)		
Democrat	1.00 (ref)	
Independent	0.79	0.50–1.26
Republican	0.49	0.34–0.71
Social ideology (n = 696)		
Moderate	1.00 (ref)	
Liberal	0.92	0.60–1.42
Conservative	0.46	0.31–0.68
Religious affiliation (n = 701)		
No religion/atheist/agnostic	1.00 (ref)	
Protestant, Catholic, Christian Orthodox, Jewish, Muslim, or other non-Christian religion	0.50	0.34–0.73
Frequency of attendance at religious services (n = 702)		
A few times a year to never	1.00 (ref)	
Once a month to more than once a week	0.63	0.46–0.87

1 Based on the 2019 Cornell National Social Survey.

2 OR = odds ratio.

3 95% CI based on profile likelihood.

4 Ref = reference level for calculation of odds ratio.

**Table 4.** Final multivariable logistic regression model for self-reported purchasing of the responsible antibiotic use-labeled milk compared with purchasing conventional milk in the 2019 Cornell National Social Survey

Predictor^1^	OR^2^	95% CI^3^

Survey question 1: Self-rated awareness of antibiotic resistance-related issues		
Not at all aware	1.00 (ref^4^)	
Any other level of awareness (somewhat aware/very aware)	2.22	1.50–3.29
Demographic variables		
Age	0.97	0.96–0.98
Education level		
High school or less	1.00 (ref)	
Technical trade, vocational school after high school, or some college, or college graduate	1.79	1.20–2.67
Postgraduate training or professional	3.46	1.98–6.14
Social ideology		
Moderate	1.00 (ref)	
Liberal	0.75	0.47–1.19
Conservative	0.54	0.35–0.82

1 Sample size = 690.

2 OR = odds ratio.

3 95% CI based on profile likelihood.

4 Ref = reference level for calculation of odds ratio.

**Table 5.** Balance of covariates between treatment arms in the experimental auction^1^

Demographic variable (definition)	Group 1	Group 2	Control

Age^1^ (yr)	42.83	40.53	40.30
Gender (1 = male, 0 = female)	0.37	0.10	0.18
Number of children	0.5	0.55	0.54
Level of education (from 1 = high school graduate to 7 = PhD)	4.48	4.83	5.04
Household income ($)	97,778	88,879	81,071
Primary shopper (1 = yes, 0 = no)	0.85	0.93	0.82
Household frequency of milk purchase (from 1 = less than once a month to 5 = more than once a week)	3.56	3.83	3.25
Self-rated knowledge of antibiotics (from 1 = know nothing to 5 = expert-level)	2.48	2.24	2.21
Self-rated concern about environment (from 1 = none to 5 = very concerned)	4.11	3.86	3.64
Self-rated health consciousness (from 1 = none to 5 = very conscious)	3.67	3.24	3.36
Typical milk size purchased (1 = gallon, 2 = half-gallon, 3 = other)	1.52	1.48	1.79
Typical fat content purchased (from 1 = nonfat to 4 = whole milk)	1.85	1.62	2.18
Liking of taste and appearance of unlabeled milk (composite of 9-point Likert scales on taste and appearance ratings)	4.03	3.79	3.32
Liking of taste and appearance of responsible antibiotic use-labeled milk (composite of 9-point Likert scales on taste and appearance ratings)	3.19	3.41	2.57
Liking of taste and appearance of organic milk (composite of 9-point Likert scales on taste and appearance ratings)	3.52	3.83	2.93

1 Full demographic survey results and definitions are in the Supplemental Material (https://doi.org/10.7298/eh5g-yf61; Schell et al., 2022).

**Table 6.** Pooled analysis of treatments in the experimental auction evaluated at mean values (willingness to pay is for half-gallon of milk)

Milk label	Blind bid ($; n = 57)	Label ($; n = 55)	Labels and education ($; n = 56)	Nutrition, education, and labels ($; n = 56)

Unlabeled milk	2.16 (1.92–2.40)^1^	1.72 (1.32–2.12)	1.83 (1.56–2.10)	1.89 (1.64–2.14)
Responsible antibiotic use-labeled milk	1.98 (1.71–2.25)	1.46 (1.11–1.81)	1.87 (1.56–2.18)	1.92 (1.61–2.23)
Organic milk	1.85 (1.80–2.10)	2.29 (2.00–2.58)	2.30 (2.00–2.59)	2.25 (1.96–2.54)

1 Parentheses represent 95% CI.

**Table 7.** Market information simulation for the experimental auction evaluated at mean values (willingness to pay is for half-gallon of milk)

Milk label	Willingness to pay^1^ ($)

Unlabeled milk	1.86 (1.58–2.13)^2^
Responsible antibiotic use-labeled milk	1.92 (1.65–2.19)
Organic milk	2.24 (1.97–2.51)

1 n = 85.

2 Parentheses represent 95% CI.
